# Immediate and Quantitative Changes in Tear Film Parameters and Meibomian Gland Structures after Warm Compression and Meibomian Gland Squeezing in Meibomian Gland Dysfunction Patients and Normal Subjects

**DOI:** 10.3390/jcm11154577

**Published:** 2022-08-05

**Authors:** Hae Min Park, Won June Lee, Han Woong Lim, Yu Jeong Kim

**Affiliations:** Department of Ophthalmology, Hanyang University Hospital, Hanyang University College of Medicine, 222-1, Wangsimni-ro, Seongdong-gu, Seoul 04763, Korea

**Keywords:** interferometer, meibomian gland dysfunction, meibomian gland squeezing, tear film lipid layer, warm compression

## Abstract

Meibomian gland dysfunction (MGD), a chronic abnormality of meibomian glands, causes various dry eye symptoms. Principal treatments for MGD are warm compression and mechanical squeezing of the eyelids. In this study, the immediate impact of this treatment on tear film lipid layer thickness (TFLLT) and the meibomian gland (MG) structure in MGD and normal groups was investigated to establish its efficacy and potential side effects. Nineteen MGD patients and seven normal subjects were enrolled. TFLLT and blinking parameters were evaluated before and after warm compression. Morphological changes of MG structures after mechanical squeezing were analyzed using Image J and Fiji. Differential analysis of the MGD and the normal groups of TFLLT changes after warm compression showed a significant increase in the normal group. In normal eyes, the average, maximum, and minimum TFLLT were significantly increased, and in the MGD group, only the minimum TFLLT was improved. Blinking parameters showed no significant change in either group. Morphometric analysis showed no damages of the MG after MG squeezing. A significant increase in MG length was observed in normal eyes. Warm compression immediately increased TFLLT more significantly in the normal group than in the MGD patients. Mechanical expression is a safe therapeutic option without remarkable structural MG damages.

## 1. Introduction

Meibomian gland dysfunction (MGD) is a multi-factorial, chronic diffuse abnormality of the meibomian glands. It is characterized by terminal duct obstruction and elevated intraductal pressure, which consequently leads to various ocular irritation symptoms and inflammation of the meibomian glands (MG) [1]. Without an appropriate treatment, MGD ultimately leads to gland atrophy, with alterations of glandular secretion and its lipid composition, which causes or further aggravates dry eye disease (DED) [2,3]. Thermal eyelid massage and hygiene, with mechanical expression to minimize terminal duct obstruction and lid margin inflammation, are principal treatments for MGD [4].

The application of a warm compress to the eyelids is a standard treatment for MGD [5]. Previous studies have reported that various warming devices raise the temperature of the eyelids and increase outflow of lipid from the MG in healthy eyes or in MGD patients [6,7,8]. The melting range of expressed meibum is between 19.5 and 33.8 °C in normal individuals, while it is between 32.2 and 35.3 °C in patients with MGD [9,10,11]. Although there were some studies related to TFLLT changes after warm compression, these have involved single-group participants who were either normal or had MGD [12,13]. According to our literature review, no previous studies reported on the quantitative comparison of tear film parameters, specifically TFLLT, between normal and MGD groups.

Forceful expression of the meibomian glands to remove obstructive material has been reported as a treatment for obstructive MGD. Interestingly, its possibly unfavorable effects have not been reported yet. Korb et al. have measured the pressure required to express meibomian glands [14]. They reported that 10 to 40 pounds per square inch (psi) were required for therapeutic MG expression and that 5 to 40 psi were required to express the first non-liquid material for any gland. Translating these pressure values into real-life examples, 80 psi corresponds to the air pressure of a bike tire, which is not negligible. Due to the atrophy of the MG in MGD, mechanical pressure for therapeutic purposes might cause unexpected damages.

Thereby, the aim of this study was to quantitatively evaluate immediate effects of warm compression on various tear film parameters in normal and MGD groups. We also evaluated MG structures before and shortly after MG expression treatments in order to assess structural MG damages related to direct pressure from mechanical expression.

## 2. Materials and Methods

Participants were recruited in this prospective study between September 2019 and March 2020 at the Department of Ophthalmology, Hanyang University Hospital, Korea. This study adhered to the tenets of the Declaration of Helsinki. It was approved by the Ethical Committee and Institutional Review Board (IRB) of Hanyang University Hospital (IRB No. 2018-11-025).

A total of 26 eyes of 26 patients were enrolled in this study, according to the inclusion criteria. Of these 26 subjects, 19 were MGD patients, and 7 were normal controls. Subjects were diagnosed with MGD by a single examiner (Y.J.K.), according to the following criteria: the presence of ocular symptoms, at least one lid margin abnormality, and impaired meibum quality or expression [15]. Inclusion criteria were: adult patients over 20 years old who gave informed consent to participate in this study. Exclusion criteria were: the use of topical medications for eye diseases other than DED, use of contact lenses within one month, history of prior ophthalmologic surgeries within one year, TFLLT values higher than 100 ICU, and conformance factor less than 0.7. The right eye was chosen for analysis.

All enrolled patients underwent basic ophthalmologic evaluations and clinical assessment of MGD by a single specialist (Y.J.K). Lid margin findings of both the upper and lower eyelids were evaluated with a slit-lamp microscope [15]. Meibomian gland secretion quality and expressibility were assessed on a scale of 0 to 3 [1]. The sum of values for upper and lower eyelids was used.

All patients received warm compression using a moist, warm compression (Bruder, The Dry Eye Doctor, Inc., Cumming, GA, USA), according to the manufacturer’s instruction. The Bruder warm compression mask was microwaved for 20–25 s and applied over the eyelids for 10 min. The measurement of TFLLT via interferometer (Lipiview, TearScience^®^ Inc., Morrisville, NC, USA) was performed sequentially both before and after warm compression. Following warm compression, mechanical MG expression was performed by the above-mentioned specialist (Y.J.K), using an Arita Meibomian Gland Compressor (Katena Inc., Denville, NJ, USA). Dynamic meibomian imaging (DMI) with a noncontact meibography device (Lipiview, TearScience^®^ Inc., Morrisville, NC, USA) was performed at the baseline and after mechanical MG expression. Meibography was performed for both the upper and lower eyelids.

An interferometer (Lipiview, TearScience^®^ Inc., Morrisville, NC, USA) was used to evaluate the tear lipid characteristic of the lower 1/3 region of the cornea. TFLLT was calculated as the minimum, maximum, and average values with standard deviation. Total blink frequency (TBF), partial blink frequency (PBF), and complete blink frequency (CBF) were also recorded with the device. Using these parameters, the partial blink rate (PBR) was calculated by dividing the PBF with the CBF. The upper cut-off value of TFLLT measurable in Lipiview was 100 ICU.

To evaluate MG damage following mechanical expression, noncontact meibography was performed for the upper and lower eyelids of both eyes, with a Meibomian Gland Evaluator (Lipiview, TearScience^®^ Inc., Morrisville, NC, USA). The partial gland was evaluated on a scale of 0 to 3, and the gland dropout was assessed on a scale of 0 to 2 [15]. Values for the upper and lower eyelids were added to give a maximal total score, which was then used for statistical analysis.

**Figure 1:**
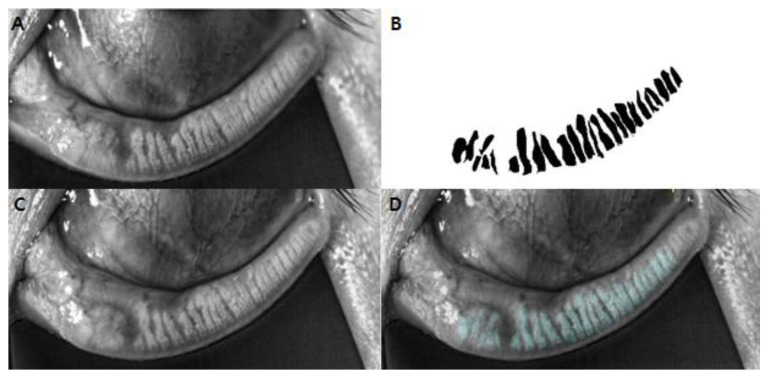
**The** representative example of overlaid, dynamic meibomian images (DMI) using Fiji. (**A**) Anonymized DMI of lower eyelid before mechanical expression. (**B**) Binary mask images constructed from A, by manual delineation of each meibomian glands. (**C**) Anonymized DMI of lower eyelid after mechanical expression. (**D**) Overlay of the mask image of pre-expression (**B**) onto the post-expression DMI image (**C**).

Morphometric analysis was conducted with Image J (National Institute of Health and the Laboratory for Optical and Computational Instrumentation, Bethesda, MD; Madison, WI, USA). DMI data were loaded into the software. Each meibomian gland of the entire eyelid was manually delineated to measure its thickness and length. Furthermore, to quantitatively evaluate miniscule gland structure changes, baseline and post-mechanical compression DMI images were manually annotated and overlaid using Fiji (fiji.sc). Detailing the process, one of the authors (H.M.P) manually marked each meibomian gland using a polygon and made annotated, masked images of pre-mechanical compression DMI images, which were then turned into binary images. These binary images of pre-mechanical compression were overlaid onto the post-mechanical compression images using Fiji [16]. Specific eyelid characteristics were regarded as landmarks to ensure comparison of MGs at the same locus. Alterations of structural shape, size, and atrophy of meibomian glands following mechanical expression were manually and meticulously evaluated by two examiners (Y.J.K, H.M.P). Examples of image processing and overlaid images are presented in Figure 1.

All statistical analyses were performed using SPSS version 22.0 (SPSS Inc., Chicago, IL, USA). All presented data are described as means ± standard deviations. Wilcoxon tests were used to analyze changes of parameters from the baseline in the normal and the MGD groups, respectively. The same test was also performed for the entire data set. Variables between the normal and the MGD groups were compared using the Mann–Whitney U test. *p* values < 0.05 were considered statistically significant.

## 3. Results

A total of 26 patients with 26 eyes were included in this study. There were 19 eyes in the MGD group and 7 eyes in the control group. Demographic data and clinical meibomian gland gradings are described in Table 1. There was no significant difference in age and sex between the MGD group and the control groups (*p* = 0.480 and 0.083). Comparison of the two groups demonstrated statistically significant differences in eyelid vascularity (*p* < 0.01), plugging (*p* < 0.01), and meibomian gland expressibility (*p* = 0.005) between the two groups. Others showed no statistically significant differences between the two groups.

The entire included data set was analyzed for TFLLT and interferometer-derived parameters before and after warm lid compression. Results are shown in Table 2. Statistical analysis showed that the average TFLLT (62.04 ± 22.68 to 82.28 ± 20.83, *p* = 0.002), maximum TFLLT (78.12 ± 22.75 to 90.81 ± 13.71, *p* = 0.016), and minimal TFLLT (54.31 ± 21.20 to 76.38 ± 23.09, *p* = 0.001) were all significantly increased after warm lid compression. However, the TBF, CBF, PBF, and PBR showed no significant change after warm compression.

When the TFLLT and the blinking parameters were analyzed separately for normal subjects and MGD patients, definite improvement of the TFLLT was observed in the normal group. (Table 3) After warm lid compression, the average, maximum, and minimum TFLLTs were all improved in the normal group, whereas only the minimum TFLLT showed improvement in the MGD group. In the normal group, the average, maximum, and minimum TFLLTs were improved from 54.71 ± 20.25 to 96.14 ± 7.58 (*p* = 0.018), 74.57 ± 21.85 to 100 ± 0 (*p* = 0.028), and 46.29 ± 13.76 to 90.29 ± 10.10 (*p* = 0.018), respectively. The minimum TFLLT in the MGD group was improved from 57.26 ± 22.96 to 71.26 ± 24.57 (*p* = 0.042). There was no statistically significant change in blink frequency or PBR after warm lid compression in either group.

From an analysis of the entire data set, the lengths of meibomian glands were increased from 4.45 ± 0.74 mm to 4.60 ± 0.90 mm (*p* = 0.047). Partial gland, gland dropout, and MG thickness did not show statistically significant change after mechanical compression (Table 4).

Table 5 shows the differential analysis of MG morphology for the MGD and normal groups. In the MGD group, the MG length increased from 4.08 ± 0.44 mm to 4.20 ± 0.78 mm, although such increase was statistically insignificant (*p* = 0.401). On the contrary, statistically significant length elongation was observed in the normal group. The MG lengths in the normal groups were increased from 4.87 ± 0.82 mm to 5.06 ± 0.86 (*p* = 0.028). A qualitative comparison of overlaid DMI images showed similar results. The MG length was increased, whereas partial gland, gland dropout, and MG thickness showed no significant changes (Figure 1). No structural damages after mechanical expression were observed.

## 4. Discussion

Among the three tear film layers, the outer lipid layer is thought to retard evaporation and increase tear film stability [17]. MGD can reduce TFLLT and alter its distribution, consequently leading to dry eye symptoms, ocular irritation, and many other symptoms. Warm compression, eyelid hygiene, and mechanical squeezing are usually recommended for MGD treatment [5,18]. Multiple studies have reported long-term effectiveness of warm lid compression and mechanical expression in treating MGD [18,19]. However, immediate effects of such treatments and comparisons of those effects between the normal group and the MGD group have not been reported. Thus, this study evaluated immediate and quantitative effects of warm lid compression and mechanical expression on the TFLLT, blinking parameters, and meibomian gland structures in normal subjects and MGD patients.

In MGD, the meibum is altered to consist of lower levels of unsaturated fatty acids and non-polar lipids [20,21], raising the melting point and causing the lipid secretions to solidify. The application of warm compression can raise the temperature of the lids beyond the meibum melting point and increase the outflow of lipid from glands and consequently, prevent water evaporation from the ocular surface, thus improving tear film quality [22,23].

Our results showed that warm lid compression alone could induce an immediate increase in TFLLT in normal patients, whereas such increases in MGD patients were minimal. This limited increment of TFLLT in MGD eyes might be attributable to the melting point and turbidity of meibum. In MGD eyes, the meibum has a higher melting point and becomes more turbid than in normal eyes [24,25]. Therefore, warm lid compression alone may be insufficient for the adequate melting of the meibum in MGD eyes. Besides meibum melting, the degree of MG atrophy might have also affected our results. MGD patients had fewer expressible glands than normal subjects. Thus, their increase in TFLLT after warm compression would have been less.

In this current study, qualitative assessment of meibomian glands with DMI revealed no remarkable damages from mechanical MG expression. Instead, the length of the MGs increased after treatment in normal eyes. Such an increase of MG length might be related to the flexibility of acinar. Unlike normal eyes, the decreased flexibility of acinar in MGD eyes due to high, intraductal pressure and consequent fibrosis or atrophy of glands may have led to insignificant changes in MG length or thickness. Except for an increase in MG length in normal subjects, there was no obvious MG damage after treatment in the normal or the MGD group. This indicates that mechanical squeezing can be safely performed without adverse effects in MGD eyes of rather severe grading. However, further study will be needed to evaluate its long-term effect.

This study has some limitations. First, it had a small sample size, especially that of the normal participants. During the enrollment process, it was very difficult to recruit normal subjects because meibomian gland squeezing is quite painful and discomforting. A sufficient and balanced sample size will be required for future research. Second, morphometric analysis of DMI was time consuming and subjective. We tried to reduce the bias by using the average value measured by two examiners. Finally, confounding variables, parameters of subjective symptoms, and anterior segment examination were not analyzed in this study.

## 5. Conclusions

In conclusion, warm compression alone can increase TFLLT in both the normal and the MGD patients, but had minimal effects on the MGD patients. In both the MGD and normal patients, no immediate structural MG damages were observed following mechanical expression. Although its long-term effect should further be studied, warm compression in conjunction with mechanical expression is a safe and effective therapeutic option without remarkable structural MG damages.

## Figures and Tables

**Table 1:** Demographics and clinical characteristics of the participants.

	All (N = 26)	MGD (N = 19)	Normal (N = 7)	*p*-Value
Age	38.53 ± 14.02	42.08 ± 15.86	30.83 ± 0.41	0.480
Sex (male:female)	8:18	4:15	4:3	0.083
Eyelid				
Vascularity	2.78 ± 1.99	4.0 ± 1.34	0.86 ± 1.07	<0.01 *
Plugging	1.50 ± 1.2	2.27 ± 0.65	0.26 ± 0.76	<0.01 *
Irregularity	0.11 ± 0.32	0.18 ± 0.41	0.0 ± 0.0	0.245
Thickening	0.56 ± 0.92	0.91 ± 1.04	0.0 ± 0.0	0.041
Meibum quality	2.73 ± 1.42	3.07 ± 0.96	2.00 ± 2.00	0.055
Meibum expressibility	3.09 ± 1.90	3.87 ± 1.72	1.43 ± 0.98	0.005 *

The data are expressed as means ± standard deviation or no. * indicating a *p*-value < 0.05. MGD, meibomian gland dysfunction.

**Table 2:** Tear film lipid layer thickness and interferometer-derived parameters before and after warm compression treatment. (Analysis with the entire data set).

		Before Treatment	After Treatment	*p*-Value
Tear Film Lipid Layer Thickness (TFLLT)				
	Average	62.04 ± 22.68	82.27 ± 20.83	0.002 *
	Maximum	78.12 ± 22.75	90.81 ± 13.71	0.016 *
	Minimum	54.31 ± 21.20	76.38 ± 23.09	0.001 *
Total blink frequency (TBF)		5.31 ± 5.28	4.85 ± 4.90	0.407
Complete blink frequency (CBF)		2.27 ± 4.51	2.19 ± 4.89	0.923
Partial blink frequency (PBF)		3.04 ± 2.24	2.65 ± 2.42	0.613
Partial blink rate (PBR)		0.67 ± 0.38	0.64 ± 0.39	0.943

The data are expressed as means ± standard deviation. * indicating a *p*-value < 0.05.

**Table 3:** Comparison of tear film lipid layer thickness and interferometer-derived parameters before and after treatment. (Differential analysis for MGD and normal groups).

		MGD			Normal		
		Before Tx	After Tx	*p*-Value	Before Tx	After Tx	*p*-Value
TFLLT							
	Avr	64.74 ± 23.43	77.16 ± 21.93	0.067	54.71 ± 20.25	96.14 ± 7.58	0.018 *
	Max	79.42 ± 23.51	87.42 ± 14.71	0.164	74.57 ± 21.85	100 ± 0	0.028 *
	Min	57.26 ± 22.96	71.26 ± 24.57	0.042 *	46.29 ± 13.76	90.29 ± 10.10	0.018 *
TBF		5.63 ± 6.05	5.16 ± 5.61	0.568	4.43 ± 2.30	4.00 ± 2.08	0.396
CBF		3.00 ± 5.11	2.58 ± 5.57	0.575	0.29 ± 0.49	1.14 ± 2.19	0.18
PBF		2.63 ± 2.22	2.58 ± 2.67	0.95	4.14 ± 2.04	2.86 ± 1.68	0.279
PBR		0.56 ± 0.40	0.57 ± 0.40	0.285	0.95 ± 0.10	0.81 ± 0.32	0.592

The data are expressed as means ± standard deviation. * indicating a *p*-value < 0.05. Avr, Average; CBF, complete blink frequency; Max, Maximum; Min, Minimum; MGD, meibomian gland dysfunction; PBF, partial blink frequency; PBR, partial blink rate; TBF, Total blink frequency; TFLLT, tear film lipid layer thickness; Tx, treatment.

**Table 4:** Meibography before and after mechanical compression. (Analysis with entire dataset).

	Before Treatment	After Treatment	*p*-Value
Partial glands	2.27 ± 1.58	2.20 ± 1.57	0.317
Gland dropout	1.93 ± 0.96	1.93 ± 0.96	1
Length (mm)	4.45 ± 0.74	4.60 ± 0.90	0.047 *
Thickness (mm)	0.74 ± 0.15	0.74 ± 0.15	0.814

The data are expressed as means ± standard deviation. * indicating a *p*-value < 0.05.

**Table 5:** Meibography before and after mechanical compression. (Differential analysis by MGD and normal groups).

	MGD			Normal		
	Before Tx	After Tx	*p*-Value	Before Tx	After Tx	*p*-Value
Partial glands	3.38 ± 1.06	3.25 ± 1.17	0.317	1.0 ± 1.0	1.0 ± 1.0	1
Glands dropout	2.38 ± 0.74	2.38 ± 0.74	1	1.43 ± 0.98	1.43 ± 0.98	1
Length (mm)	4.08 ± 0.44	4.20 ± 0.78	0.401	4.87 ± 0.82	5.06 ± 0.86	0.028 *
Thickness (mm)	0.71 ± 0.16	0.69 ± 0.19	0.31	0.79 ± 0.13	0.81 ± 0.11	0.345

The data are expressed as means ± standard deviation or no. * indicating a *p*-value < 0.05. MGD, meibomian gland dysfunction; Tx, treatment.

## Data Availability

The data presented in this study are available on request from the corresponding author. The data are not publicly available due to restrictions eg privacy or ethical.
